# PD-L1 as a Novel Mediator of Lung Fibroblast to Myofibroblast Transition

**DOI:** 10.33696/immunology.4.142

**Published:** 2022

**Authors:** Xia Guo, Guoqing Qian

**Affiliations:** Department of Cellular and Molecular Biology, The University of Texas Health Science Center at Tyler, Tyler, TX, USA

**Keywords:** Fibroblast to myofibroblast transition, Lung, PD-L1, Pulmonary fibrosis, Transforming growth factor β

Idiopathic pulmonary fibrosis (IPF) is an incurable disease that affects approximately 3 million people worldwide [1]. Although two drugs (nintedanib and pirfenidone) have been approved for treatment of IPF, currently there is a lack of effective pharmacotherapy that could stop the progression or offer a cure for this devasting disease.

Programmed death-ligand 1 (PD-L1) is an immune checkpoint protein that serves as a mechanism of immune surveillance. It limits excessive immune response through binding with its receptor PD-1 on T cells and suppressing their activation [2]. Increasing evidence also unveils an important role of PD-L1 in cancer cell proliferation, metastasis, and drug resistance to targeted therapy [2,3]. Recently, a few papers published support targeting PD-L1/PD-1 axis for treatment of IPF [4-7]. The treatment with an anti-PD-L1 antibody attenuates/blocks the progression of pulmonary fibrosis in preclinical models of IPF. Beyond the immune cells, new evidence has also suggested a novel role of PD-L1 in fibroblasts [5,8], particularly through a process termed fibroblast to myofibroblast transition (FMT) or myofibroblast differentiation.

Lung fibroblasts play a critical role in the progression of pulmonary fibrosis. One critical feature lies in the differentiation of fibroblasts to myofibroblasts, which express structural protein α-SMA and excessive extracellular matrix, thus contributing to the structural remodeling of the lung. Myofibroblasts also entails positive feedback through secretion of profibrotic mediators, especially TGF-β1, the most potent inducer of profibrotic changes. Geng et al. identified a subgroup of invasive lung fibroblasts that possess high levels of PD-L1. Those fibroblasts with high PD-L1 expression can induce fibrotic changes in the mouse lungs upon tail vein injection [6]. The finding implicates PD-L1 in the development of pulmonary fibrosis *in vivo*. Kang et al. reported that TGF-β1 induces PD-L1 expression using established human and murine fibroblast cell lines and PD-L1 is implicated in FMT [5]. However, the underlying mechanisms remain elusive.

The recent publication by our group [8] provides further insight on how PD-L1 mediates FMT and how it is intervened with the TGF-β signaling. In this manuscript, the authors first demonstrated an enhanced expression of PD-L1 in the fibrotic lungs of IPF patients and mice models of pulmonary fibrosis. The finding provides a proof of concept of the implication of PD-L1 in pulmonary fibrosis. The authors further showed that TGF-β1 induces the expression of PD-L1 in several lines of primary lung fibroblasts from both normal and IPF donors. These data, together with a previous report using established cell lines [5], strengthen a role of PD-L1 induction by TGF-β1 in the FMT process. In addition, the expression of PD-L1 has also been identified in alveolar/bronchial epithelial cells [9] and alveolar macrophages [10] of IPF patients, implying various sources of PD-L1 in the pathogenesis of IPF.

Both canonical and non-canonical pathways of TGF-β1 signaling contribute to the FMT process and pulmonary fibrosis development. The manuscript demonstrated that PD-L1 is involved in different pathways to promote FMT. The interaction between PD-L1 and Smad3 was confirmed through immunofluorescent co-staining of PD-L1 and phosphor-Smad3 and co-immunoprecipitation (co-IP) assay. The interaction increased with the treatment of TGF-β1, although a basal level of interaction was also found. It remains unclear, however, whether such increased interaction (co-IP) is due to elevated expression of PD-L1 or perhaps a result of increased binding affinity. Further study is also warranted to examine the transport of PD-L1 from cytoplasm to the nucleus where co-staining of PD-L1 and phosphor-Smad3 was noted. The recent identification of the p300 as the acetyltransferase of PD-L1 provides a mechanism for the nuclear transport of PD-L1 in cancer cells [11], which deserves further investigation in the setting of lung fibroblasts, especially in view of the interactions between TGF-β and p300 [12,13].

Whether the binding between PD-L1 with Smad3 also stabilizes phosphor-Smad3 and thereby increasing its activity needs to be addressed in future studies. Another shortcoming of the study is the overlook of Smad2 in the FMT process. Whether PD-L1 also binds with Smad2 to form a tri-complex with Smad3 deserves further investigation. Nevertheless, this manuscript presents novel findings that help explain how PD-L1 is intervened with the Smad3 signaling. This finding might be of general interest in terms of the complex role of TGF-β in tumor progression and tissue fibrosis of other organs. Another mechanism reported in this manuscript involves the modulation of the GSK3β/β-catenin signaling by PD-L1. Previous studies have reported that Wnt/β-catenin signaling upregulates PD-L1 transcription in diverse cancer cells [14-16]. Interestingly, PD-L1 has also been found to activate β-catenin signaling in different cancer cell types [14,17], suggesting potential positive feedback, β-catenin is a known target of GSK3β for ubiquitin dependent degradation. It is also implicated in lung myofibroblast differentiation [18]. The link between PD-L1 and β-catenin signaling provides an additional layer of control over the FMT process in the context of IPF.

The identification of the upstream regulator of PD-L1 by TGF-β provides novel insight on the control of PD-L1 levels. Interestingly, Smad3 inhibition by a specific small molecule inhibitor SIS3 attenuates the induction of PD-L1 by TGF-β. It seems that there exists a positive loop between Smad3 and PD-L1, i.e., Smad3 upregulates PD-L1 level which in turn binds to and increase the activity of Smad3. However, more experiments are needed to test this hypothesis. On the other hand, p38 pathway as a critical non-canonical TGF-β signaling has also been implicated in the regulation of PD-L1. Inhibition of p38 also attenuates the induction of PD-L1 by TGF-β. Beyond IPF, asthma related subepithelial fibrosis also benefits from the inhibition of the p38 pathway [19]. Whether targeting PD-L1 will likewise benefit fibrosis of other organs remains to be explored.

The transcriptional regulation of PD-L1 was demonstrated through an array of assays in this manuscript. It is well known that multiple mechanisms control the level of PD-L1 including manipulating its protein stability [20], e.g., the identification of March8 as a novel E3 ligase for PD-L1 degradation [21].Whether TGF-β signaling also affects the stability of PD-L1 remains an interesting question and warrants further experimental testing. The authors put together these findings nicely into a diagram. TGF-β induces PD-L1 expression that is dependent on Smad3 and p38 signaling. Increased PD-L1 in turn binds to Smad3 to enhance its activation and transcriptional activity (via α-SMA luciferase reporter assay). In addition, PD-L1 also contributes to upregulated β-catenin levels induced by TGF-β, which is likely mediated through GSK3β pathway. Through those two mechanisms, PD-L1 is implicated in TGF-β induced FMT process and potentially the development of pulmonary fibrosis. A schematic presentation of the mechanisms by which PD-L1 mediates FMT is shown in Figure 1 (Created with BioRender.com).

As mentioned above, PD-L1 expression has been reported in diverse cell types including alveolar macrophages [10], alveolar epithelial cells [9,22], and lung fibroblasts [4-6,8]. Therefore, the generation of fibroblast-specific mouse model is a prerequisite for testing the role of PD-L1 in lung fibroblasts towards pulmonary fibrosis development. Currently, the conditional model is not available and future work is needed to generate the fibroblast-specific PD-L1 knockout mouse model. A recent study [23] has identified a subpopulation of alveolar type II epithelial cells (AECII) that express PD-L1 and expand in the lung of IPF patients. Due to a progenitor role of these cells, a concern is raised that PD-L1 inhibition might injure the already compromised epithelial compartment in the IPF lung. Similarly, this speculation remains to be tested using conditional PD-L1 knockout mice model.

In summary, the immune checkpoint protein PD-L1 has emerged as a novel target for the treatment of IPF. Accumulating evidence supports the targeting of PD-L1 to block the development of pulmonary fibrosis. Experiments are needed, however, to test whether inhibition of PD-L1 could reverse the already existent fibrotic lesions in the lung of IPF patients, which is often the case in the clinic. It is likely that the role of PD-L1 in the progression of pulmonary fibrosis is complex, arising from diverse cell types and involving different mechanisms. The generation of cell-specific PD-L1 knockout animal models might hold the key to unveil the full view of PD-L1 in the development of IPF.

## Figures and Tables

**Figure 1:**
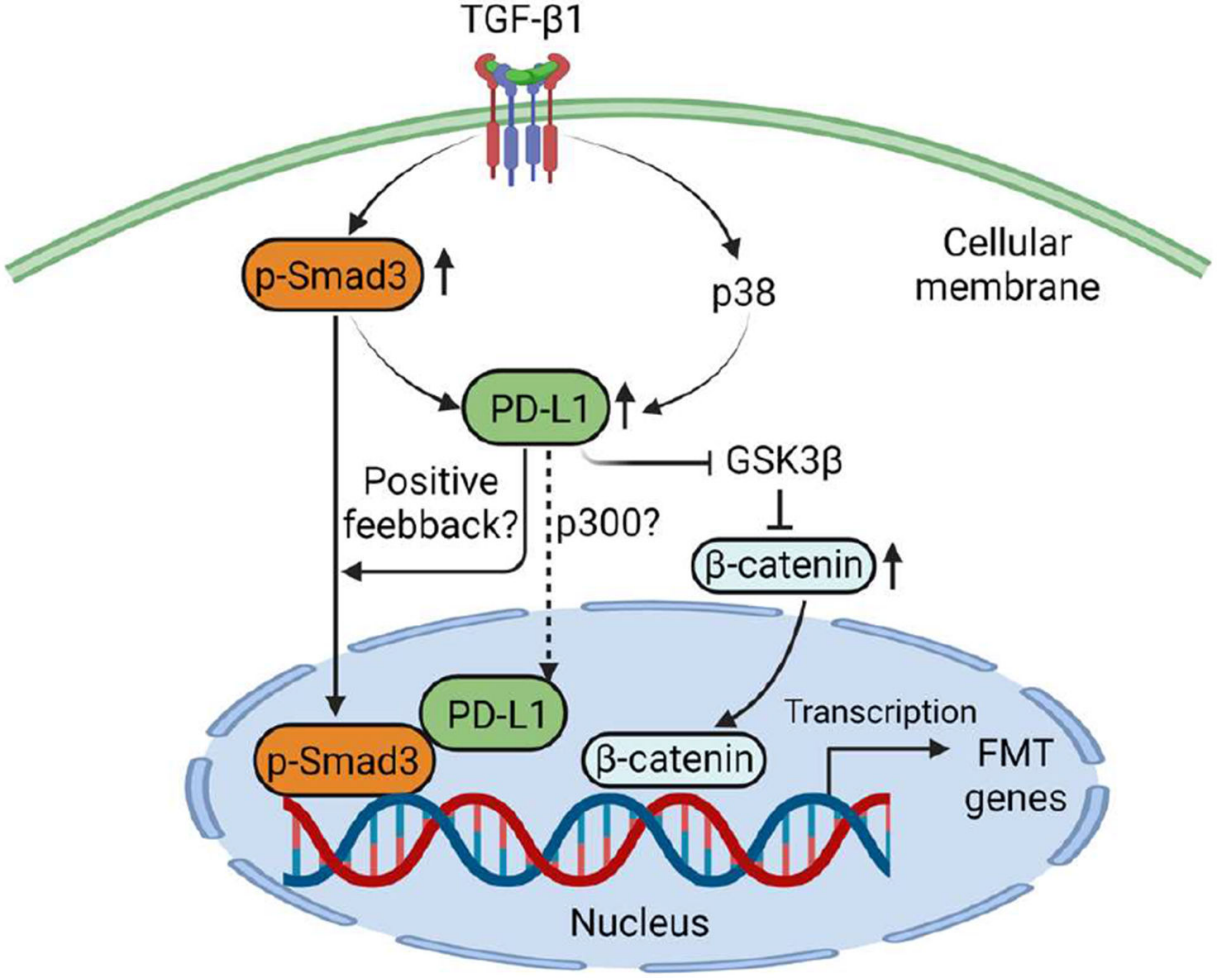
A diagram of the mechanisms by which PD-L1 mediates fibroblast to myofibroblast transition (FMT).
